# Propofol enhances BCR-ABL TKIs’ inhibitory effects in chronic myeloid leukemia through Akt/mTOR suppression

**DOI:** 10.1186/s12871-017-0423-2

**Published:** 2017-09-29

**Authors:** Zhimin Tan, Aixia Peng, Jingwen Xu, Mingwen Ouyang

**Affiliations:** 1 Department of Anesthesiology, Shenzhen Hospital, Southern Medical University, Xinhu Road No.1333, Bao’an district, Shenzhen, 518100 Guangdong province China; 2 Department of Oncology, Shenzhen Hospital, Southern Medical University, Xinhu Road No.1333, Bao’an district, Shenzhen, 518100 Guangdong province China; 3 Department of Anesthesiology, Fifth Affiliated Hospital, Southern Medical University, Congcheng Road No. 566, Conghua district, Guangzhou, Guangdong province 510900 China

**Keywords:** Leukemia, Propofol, Akt/mTOR, Drug repurposing

## Abstract

**Background:**

The anti-cancer activities of intravenous anesthetic drug propofol have been demonstrated in various types of cancers but not in chronic myeloid leukemia (CML).

**Methods:**

We systematically examined the effect of propofol and its combination with BCR-ABL tyrosine kinase inhibitors (TKIs) in CML cell lines, patient progenitor cells and mouse xenograft model. We analyzed propofol’s underlying mechanism focusing on survival pathway in CML cells.

**Results:**

We show that propofol alone is active in inhibiting proliferation and inducing apoptosis in KBM-7, KU812 and K562 cells, and acts synergistically with imatinib or dasatinib, in in vitro cell culture system and in vivo xenograft model. In addition, propofol is more effective in inducing apoptosis and inhibiting colony formation in CML CD34 progenitor cells than normal bone marrow (NBM) counterparts. Combination of propofol and dasatinib significantly eliminates CML CD34 without affecting NBM CD34 cells. We further demonstrate that propofol suppresses phosphorylation of Akt, mTOR, S6 and 4EBP1 in K562. Overexpression of constitutively active Akt significantly reverses the inhibitory effects of propofol in K562, confirm that propofol acts on CML cells via inhibition of Akt/mTOR. Interestingly, the levels of p-Akt, p-mTOR and p-S6 are lower in cells treated with combination of propofol and imatinib than cells treated with propofol or imatinib alone, suggesting that propofol augments BCR-ABL TKI’s inhibitory effect via suppressing Akt/mTOR pathway.

**Conclusion:**

Our work shows that propofol can be repurposed to for CML treatment. Our findings highlight the therapeutic value of Akt/mTOR in overcoming resistance to BCR-ABL TKI treatment in CML.

**Electronic supplementary material:**

The online version of this article (10.1186/s12871-017-0423-2) contains supplementary material, which is available to authorized users.

## Background

Chronic myeloid leukemia (CML) is a hematological stem cell malignancy. The majority of CML are due to transformation of oncogene BCR-ABL and 1–2% CML are BCR-ABL negative [1, 2]. Treatment with tyrosine kinase inhibitors (TKIs) specifically targeting BCR-ABL by binding to the ATP-binding site of Abl, such as imatinib and dasatinib, results in significant improvement in clinical responses of CML patients [3, 4]. However, patients achieving remission with BCR-ABL TKIs continue to have molecular evidence of persistent disease and major mechanisms are due to Bcr-Abl protein overexpression and mutations [5]. Other BCR-ABL-independent resistance mechanisms have been identified to be compensatory activation of phosphoinositide 3-kinase (PI3K)/Akt/mammalian target of rapamycin (mTOR) and Wnt/β-catenin, and suppression of protein phosphatase 2A [6–8]. Therefore, identification of compounds that target the molecules involved in the resistance may provide an alternative therapeutic strategy for CML treatment.

Propofol is a general sedative reagent and commonly used for induction and maintenance of general anesthesia [9]. It has advantages over other anesthetic drugs by protecting neuron and endothelial cells from oxidative stress and hypoxia injury [10, 11]. Interestingly, increasing studies have demonstrated that propofol inhibits the growth, migration and invasion and induces apoptosis of tumor cells of diverse tissue origins, such as ovarian, cervix, lung and gastric-intestinal tract [12–16]. The synergistic effects of propofol with conventional chemotherapeutic drugs have been demonstrated in cervical and ovarian cancer cells [13, 17]. The mechanism of action of propofol in cancer is not completely understood and seems to be different in various tumor types. For example, it kills lung cancer cells via inducing endoplasmic reticulum stress [16] whereas promotes cervical cancer cell apoptosis via inhibiting mTOR pathway [18].

In this study, we examined the effect of propofol alone and its combinatory effect with BCR-ABL TKIs in CML cell lines, primary CD34 progenitor cells and xenograft mouse model. We show that propofol is effective in targeting multiple aspects of CML cells and acts synergistically with BCR-ABL TKIs in vitro and in vivo. We further show that propofol augments TKIs’ effect via suppressing Akt/mTOR signaling pathway in CML cells.

## Methods

### CML patient CD34 cells, cell lines and drugs

CD34 cells were obtained from tissue repository in Shenzhen Hospital of Southern Medical University and The Fifth Affiliated Hospital of Southern Medical University. Human normal bone marrow (NBM) CD34 progenitor cells were purchased from LONZA Group. CD34 cells were cultured in a serum-free medium supplemented with multiple recombinant cytokines for myelopoiesis of hematopoietic progenitor cells as previously described [19]. Human CML cell lines (eg. K562, KU812 and KBM-7) were purchased from American Type Culture Collection and cultured in RPMI1640 medium supplemented with 10% fetal bovine serum and 2 mM L-glutamine. Dasatinib (LC laboratories, US) and propofol (Sigma, US) were reconstituted in dimethyl sulfoxide (DMSO) and imatinib (Sigma, US) was reconstituted in water.

### MTS proliferation assay

Equal number of CML cells (10,000) were seeded into 96-well-plate and incubated with propofol or imatinib alone or combination of propofol and imatinib for 72 h. Cell proliferative activity was then measured by using CellTiter 96® Aqueous One Solution Cell Proliferation Assay kit (Promega, US) according to manufacture’s instruction.

### Apoptosis analysis and caspase-3activity assay

CML cells (500, 000) were seeded into 12-well-plate and incubated with propofol or imatinib alone or combination of propofol and imatinib for 72 h. Apoptotic cells were labeled by Annexin V-FITC and 7-AAD staining using ANNEXIN V-FITC / 7-AAD Kit (Beckman Coulter, France). Quantification of apoptotic cells were achieved by performing flow cytometry on MACSQuant® Analyzer (Miltenyl Biotec, US). Cells were incubated with propofol for 48 h prior to caspase 3 activity assay using Caspase 3 Assay Kit (Abcam, US).

### Colony formation

CD34 cells, HSC-CFU complete w/o Epo methylcellulose medium (Miltenyi Biotec, Germany) together with drug were mixed well and plated onto 6-well-plate. After 10–14 days, colonies were visualized under microscopy and the number of colonies was scored. Clusters with more than 100 cells were counted as a colony.

### Western blot

K562 cells (2,000,000) were seeded into 6-well-plate and incubated with propofol or imatinib alone or combination of propofol and imatinib for 24 h. Treated cells were then lysed in M2 lysis buffer (20 mM Tris at pH 7, 0.5% NP-40, 250 mM NaCl, 3 mM EGTA, 3 mM EDTA, 2 mM dithiothreitol, 20 mM glycerol phosphate, proteinases inhibitor cocktail). Protein concentration was determined using bicinchoninic acid protein assay kit (Thermo Scientific, US). Equal amount of protein were resolved by SDS-PAGE and transferred onto PVDF membrane (Bio-Rad, US). The membrane was then analysed by western blot with designated primary and secondary antibodies. Signals were developed with the chemiluminescence kits (Amersham Biosciences, UK) and visualized with the Kodak Image Station.

### Plasmid transfection

K562 cells were transfected by treating the cells with Lipofectamine® Transfection Reagent (Thermo Scientific, US) and 2 μg Vector, or myr Akt (constitutively active Akt) plasmid (a kind gift from Dr. Richard Roth) [20] using the protocol provided by the manufacture. At 24 h post-transfection, cells were used for rescue experiments.

### CML xenograft in SCID mouse

Animal experiments were carried out in compliance with the Fifth Affiliated Hospital of Southern Medical University. 6-week-old male NOD/SCID mice were purchased from Hunan SJA Animal Laborator Co. Ltd. K562 cells (10, 000,000) were harvested and suspended in 100 μl cold PBS. Cells were subcutaneously injected into mice flank. After development of palpable tumor, mice were randomized into four groups and treated with intraperitoneal control (80/20%, saline/DMSO), oral dasatinib, intraperitoneal propofol or combination of dasatinib and propofol daily. Tumor size were measured every 3 days. After 3 weeks treatment, mice were euthanized and tumors were weighed.

### Statistical analyses

All experiments were repeated at least three times with similar results. The data are expressed as mean ± S.D. An unpaired Student’s t test was applied to determine statistical significance with *p* < 0.05.

## Results

### Propofol is active against CML cells and significantly augments BCR-ABL TKI’s effects

**Fig. 1:**
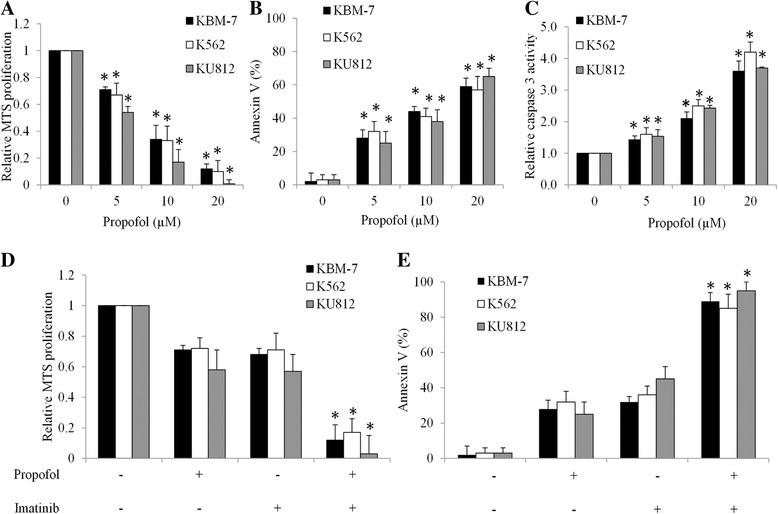
Propofol is active against CML cells and enhances imatinib’s inhibitory effects. Propofol significantly inhibits proliferation (**a**), induces apoptosis (**b**) and increases caspase 3 activities (**c**) in K562, KBM7 and KU812 cells in a dose-dependent manner. Combination of propofol and imatinib results in significant more proliferation inhibition and apoptosis induction than propofol or imatinib alone in CML cells. Propofol at 5 μM and imatinib at 1 μM were used for combination studies. DMSO (final concentration 0.5%) was used as control. *p < 0.05, compared to control or single drug alone

We examine the effect of propofol by performing cell growth and survival assays in CML cells exposed to various concentrations of propofol. A panel of human CML cell lines were used, including KBM-7, K562 and KU812. All these cell lines are derived from CML patients in blast crisis stage and in vitro models for the study of CML [21]. We show that propofol at 5, 10 and 20 μM significantly inhibits proliferation of KBM-7, K562 and KU812 cells in a dose-dependent manner (Fig. 1a). We then treated CML cells with propofol and analyzed the Annexin V level, which is a well-known marker for apoptosis [22]. As shown in Fig. 1b, propofol significantly increase Annexin V levels in CML cells. Such an effect of propofol is further confirmed by the increased level of active caspase 3 in propofol-treated cells (Fig. 1c), demonstrating that propofol induces CML cell apoptosis.

We next performed combination studies using propofol and imatinib at concentration that has only mild inhibitory effects to CML as single drug alone. The results show that propofol at 5 μM and imatinib at 1 μM alone results in ~ 30% inhibition of growth and survival in KBM-7, KU812 and K562 cells (Fig. 1d and e). In contrast, combination of both results in ~80% inhibition of growth and survival in CML cells (Fig. 1d and e).

### Propofol significantly augments BCR-ABL TKI’s effects in CML CD34 progenitors while sparing normal hematopoietic progenitors

**Fig. 2:**
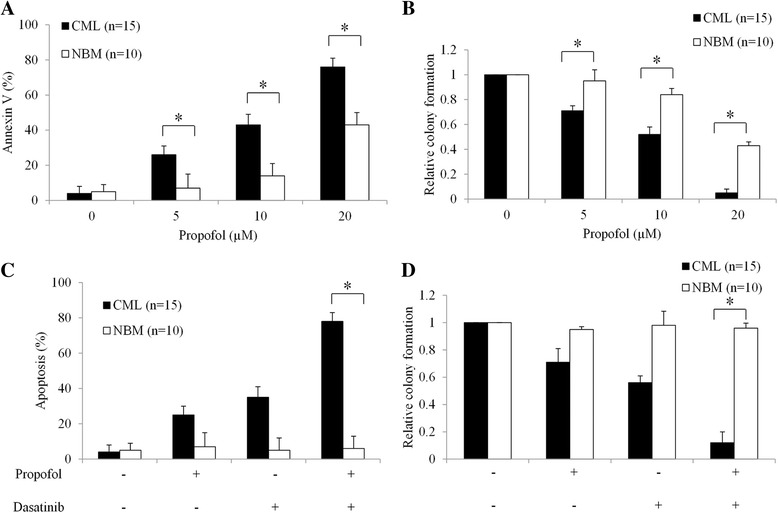
Propofol is selectively active against CML CD34 progenitor cells and enhances dasatinib’s inhibitory effects. Propofol is more effective in inducing apoptosis (**a**) and inhibiting colony formation (**b**) in CML than NBM CD34 progenitor cells. Combination of propofol and dasatinib results in significant more apoptosis induction and colony formation inhibition than propofol or dasatinib alone in CML cells. Propofol at 5 μM and dasatinib at 200 nM were used for combination studies. DMSO (final concentration 0.5%) was used as control. *p < 0.05, compared to control or single drug alone

We next examined propofol’s effect on the survival, proliferation and differentiation of CD34 progenitor cells derived from blast phase CML patients as well as normal bone marrow (NBM) CD34 cells. Fifteen CML patient and ten NBM samples were used in this study. We found that propofol at the same concentration induces more apoptosis in CML than NBM CD34 progenitor cells (Fig. 2a). Propofol is also more effective in inhibiting colony formation in CML compared to NBM CD34 progenitor cells (Fig. 2b). It is noted that combination of propofol at 5 μM and dasatinib at 200 nM significantly induces apoptosis and inhibits colony formation in CML CD34 cells without affecting NBM counterparts (Fig. 2c and d).

### Propofol acts on CML cells and enhances BCR-ABL TKI’s effect by suppressing Akt/mTOR signaling pathway

Although the anti-cancer activity of propofol has been consistently demonstrated, the molecular mechanisms of its action in cancer vary in different cancers [12, 23–25]. Essential signaling pathways involved in tumor cell proliferation and survival, such as epithelial growth factor receptor (EGFR) /Janus kinase 2 (JAK2)/ Signal transducer and activator of transcription 3 (STAT3) and mTOR pathways, have been reported to be propofol’s targets [13, 18]. In order to understand the mechanism of propofol in CML cells, we analyzed phosphorylation level of molecules involved in Akt/mTOR pathway, which is also an essential downstream pathway of BCR-ABL signaling [7].

**Fig. 3:**
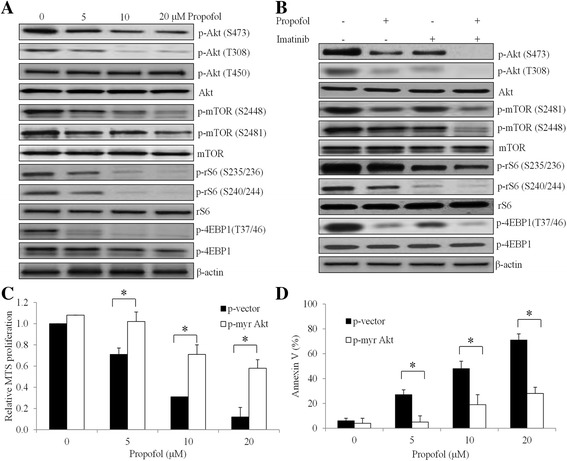
Propofol enhances imatinib’s effects in CML cells by suppressing Akt/mTOR signaling pathway. Representative western blot images showing the inhibitory effects of propofol alone (**a**) and combination of propofol and imatinib (**b**) on phosphorylation of Akt, mTOR and S6 in K562 cells. Propofol at 5 μM and imatinib at 1 μM were used for combination studies. Overexpression of constitutively active Akt (myr Akt) significantly reverses the effects of propofol in inhibiting proliferation (**c**) and inducing apoptosis (**d**) in K562 cells. DMSO (final concentration 0.5%) was used as control. *p < 0.05, compared to vector control

As shown in Fig. 3a, propofol dose-dependently suppresses phosphorylation of p-Akt at Ser473, Thr308 but not Thr450 in CML cells. Decreased levels of p-mTOR at Ser2448 and Ser2481 were observed in CML cells exposed to propofol. Consistently, phosphorylation of downstream effectors of mTOR pathway including S6, 4EBP1 were inhibited by propofol. Further decreased levels of p-Akt at Ser473 and Thr308, p-mTOR at Ser2448 and Ser2481, p-S6 at Ser235/236 and Ser240/244, and p-4EBP1 at Thr37/46 were observed in K562 cells exposed to propofol and imatinib combination compared to propofol or imatinib alone (Fig. 3b), suggesting that the combinatory effects of imatinib and propofol in CML are likely due to the further inhibition on Akt-mTOR pathway. We next generated K562 cells that overexpress myr-Akt which is anchored at the cell membrane by the myr group and thus constitutively activated by phosphatidylinositol-dependent kinase 1 [20], results in highly phosphorylated Akt that could not be inhibited by propofol (Additional file 1: Figure S1). In addition, total Akt levels are also higher in myr-Akt –expressing cells than control (Additional file 1: Figure S1). We further found that overexpression of activated Akt abolished the anti-proliferative and pro-apoptotic effects of propofol (Fig. 3c and d), confirming that propofol acts on CML cells via inhibiting Akt/mTOR pathway. To further strength our conclusion that propofol enhances BCR-ABL TKIs’ inhibitory effects in CML through Akt/mTOR suppression, we pharmacologically inhibited mTOR using rapamycin and investigated the combinatory effects of rapamycin and imatinib. Similar to propofol, we found that combination of rapamycin and imatinib was significantly more effective than single drug alone in inhibiting proliferation and inducing apoptosis in multiple CML cell lines (Additional file 1: Figure S2). This is also consistent with the previous work on the inhibitory effects of rapamycin in CML [26].

### Propofol is effective in inhibiting CML growth in vivo

**Fig. 4:**
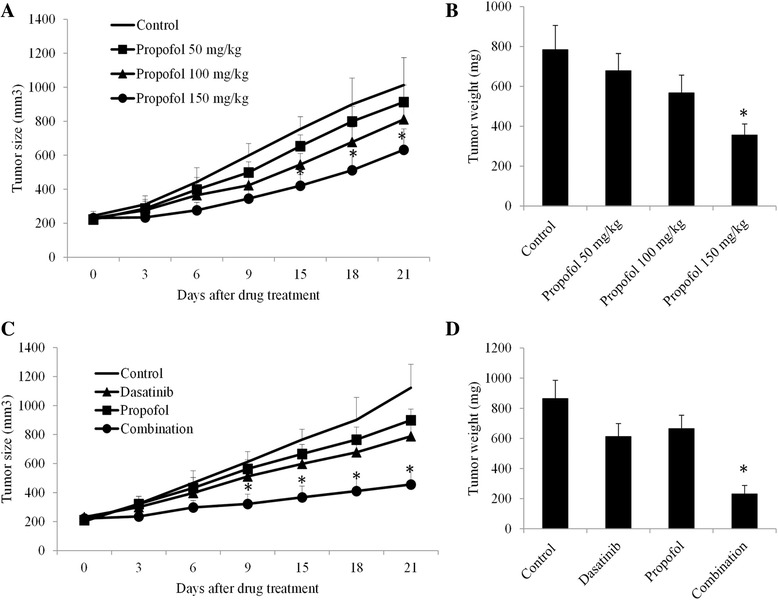
Propofol significantly inhibits CML growth in vivo and augments dasatinib’s inhibitor effect. Propofol dose-dependently decreases tumor size (**a**) and weight (**b**) in a CML xenograft mouse model. Combination of propofol and dasatinib is more effective in decreasing tumor size (**c**) and weight (**d**) than propofol or dasatinib alone. In combination studies, 20 mg/kg propofol and 5 mg/kg dasatinib was given to mice by intraperitoneal and oral administration, respectively. DMSO/Saline (20%/80%) was used as vehicle control. Tumour volume was calculated using the formula: length × width^2^/2. *p < 0.05, compared to control or single arm treatment

To investigate the in vivo efficacy of propofol alone and its combination with BCR-ABL TKI, we generated CML xenograft mouse model by subcutaneously injecting K562 cells into SCID mice flank. After development of palpable tumor, we firstly tested propofol’s efficacy as single drug alone. Propofol is a short-acting anaesthetic agent that provides rapid, smooth recovery. In mice, it has little analgesic effect [27, 28]. We observed induction, hypnosis and muscle relaxation in mice given by propofol at 60 mg/kg i.p. (the maximal dose used in the study). However, these analgesic effects last for 30 min with a fast recovery. We did not observe significant weight loss, abnormal behaviour or appearance of mice, suggesting that mice tolerate well to propofol. As shown in Fig. 4a and b, propofol at 20 and 40 mg/kg only slightly inhibited tumor growth whereas propofol at 60 mg/kg significantly reduced tumor size and weight. Consistent with in vitro data, combination of propofol and dasatinib achieved greater efficacy in inhibiting CML tumor growth than propofol or imatinib alone (Fig. 4c and d).

## Discussion

Although the inhibitory effect for an intravenous anesthetic drug propofol as an anticancer agent has been demonstrated in a panel of solid tumors [13–15, 23, 29, 30], its therapeutic potential in hematological malignancies are not well understood. CML is characterized as a hematopoietic stem cell malignancy [1]. Although imatinib or dasatinib have significantly improved clinical outcome in CML, primary resistance and leukemia relapse after an initially successful response is a major problem [31]. This suggests that addition of drug which can synergize with TKI may be better option in CML. In this work, we addressed a most relevant issue in CML patients by evaluating propofol as a potential agent for overcoming BCR-ABL TKIs resistance. Propofol is an attractive candidate as it is available in clinical practice for the induction and maintenance of general anesthesia, and have protective effects in multiple organs and tissues from hypoxia and ischemia-reperfusion injuries [11, 32]. In this study, we show that propofol is active against CML cells and significantly augments TKIs’ inhibitory effect via suppression Akt/mTOR signaling.

We used three cell lines: KBM7, K562 and KU812, which derived from different CML patients, to ensure the effect of propofol in CML. Propofol significantly inhibits proliferation, increases apoptosis and caspase 3 activation in all tested CML cell lines (Fig. 1a to c). This result is consistent with the previous reports on the inhibitory effects of propofol on the tumor cell growth and survival [13–15, 23, 29, 30]. In particular, propofol has been shown to activate caspase 3, 8 and 9 in acute myeloid leukemia cell HL60 [24]. In addition, propofol significantly sensitizes CML cell in response to imatinib (Fig. 1d and e). The plasma propofol concentrations for general anesthesia are considered to be 3–6 μg/ml [33]. The IC50 of propofol as single drug is 10 μM (1.78 μg/ml) and the dose of propofol used for combination therapy is 5 μM (0.89 μg/ml) (Fig. 1), suggesting that the effective concentrations of propofol in CML are clinically achievable. Importantly, our results obtained from CML xenograft mouse model further demonstrate the in vivo efficacy of propofol and its synergistic effects with BCR-ABL TKIs (Fig. 4). It has also been shown that propofol enhances paclitaxel- or cisplatin-induced apoptosis in ovarian and cervical cancer cells [13, 17]. Our results together with the previous studies demonstrate that propofol is a potential candidate for combination therapy in cancer treatment.

CML is recognized to be a hematopoietic stem cell disorder and CD34 cells serve as a reservoir for disease relapse [8]. In line with cell lines results, propofol also effectively induces apoptosis and inhibits colony formation of CD34 cells derived from 15 CML patients (Fig. 2a and b). Targeting tumor cells while sparing normal counterparts is critical for targeted therapy. Compared to CML CD34 cells, propofol is less effective in targeting NBM CD34 cells (Fig. 2a and b) and combination of propofol with dasatinib selectively targets CML but not NBM CD34 cells (Fig. 2c and d). The inhibitory effects of propofol in cancer are mostly demonstrated by cancer cell lines, we are the first to show propofol’s therapeutic effects in patient primary stem/progenitor cells.

We further investigated the molecular mechanism of propofol’s action in CML. It dose-dependently decreases phosphorylation levels of Akt at S473 and T308, mTOR at S2448 and S2481 in CML cells (Fig. 3a). Consistently, propofol decreases the phosphorylation of downstream effectors of mTOR pathway including S6 and 4EBP1 (Fig. 3a). Overexpression of constitutively active Akt significantly abolishes propofol’s effects in CML cells (Fig. 3c and d), confirming that propofol acts on CML via suppression Akt/mTOR pathway. Interestingly, we also find that short time exposure of CML cells to imatinib results in decreased levels of p-Akt, p-mTOR and p-S6. Further decreased phosphorylation levels of these molecules are observed in cells in the presence of both imatinib and propofol (Fig. 3b). These results suggest that propofol augments TKI’s effect by its combinatory effects in further decreasing Akt/mTOR pathway. Synergism between imatinib and inhibitors of PI3K, Akt or mTOR have been observed in CML cells [34]. In line with previous work, we demonstrate that combination of rapamycin (mTOR inhibitor) and imatinib was significantly more effective than single drug alone in inhibiting proliferation and inducing apoptosis in multiple CML cell lines (Additional file 1: Figure S2). These suggest that propofol acts similarly as mTOR inhibitors in CML. In addition, propofol has advantages over these inhibitors due to the fact that propofol has already been used in clinics.

## Conclusion

Our work demonstrate that propofol has potential to be repurposed for CML treatment given its combinatory efficacy with BCR-ABL TKI in in vitro and in vivo without affecting NBM cells. Our work also demonstrate that the combinatory efficacy are attributed to their inhibitory effects in eliminating Akt/mTOR pathway.

## Additional files

Additional file 1: Figure S1. Expression levels of p-Akt and total Akt in CML cells transfected with p-myr Akt or p-vector in the presence or absence of propofol. No significant change on Akt phosphorylation level by propofol in K562 cells transfected with constitutively active Akt plasmid. Increased expression level of Akt is shown in p-myr Akt than p-vector cells. Representative western blot photos were shown. **Figure S2.** The combinatory effects of rapamycin and imatinib in CML cells. Combination of rapamycin and imatinib results in significant more proliferation inhibition and apoptosis induction than rapamycin or imatinib alone in KBM-7, K562 and KU812 cells. Rapamycin at 1 μM and imatinib at 1 μM were used for combination studies. DMSO (final concentration 0.5%) was used as control. *p < 0.05, compared to control or single drug alone. (DOC 1147 kb)

## Abbreviations

CML: Chronic myeloid leukemia
DMSO: Dimethyl sulfoxide
EGFR: Epithelial growth factor receptor
JAK2: Janus kinase 2
mTOR: Mammalian target of rapamycin
NBM: Normal bone marrow
PI3K: Phosphoinositide 3-kinase
STAT3: Signal transducer and activator of transcription 3
TKI: Tyrosine kinase inhibitor
