# Supplementary material for: Propofol enhances BCR-ABL TKIs’ inhibitory effects in chronic myeloid leukemia through Akt/mTOR suppression
Source: BMC Anesthesiol. 2017 Sep 29;17:132. doi: 10.1186/s12871-017-0423-2 (PMC5622516; doi:10.1186/s12871-017-0423-2)
Supplement: Supplementary file 1 — Expression levels of p-Akt and total Akt in CML cells transfected with p-myr Akt or p-vector in the presence or absence of propofol. No significant change on Akt phosphorylation level by propofol in K562 cells transfected with constitutively active Akt plasmid. Increased expression level of Akt is shown in p-myr Akt than p-vector cells. Representative western blot photos were shown. Figure S2. The combinatory effects of rapamycin and imatinib in CML cells. Combination of rapamycin and imatinib results in significant more proliferation inhibition and apoptosis induction than rapamycin or imatinib alone in KBM-7, K562 and KU812 cells. Rapamycin at 1 μM and imatinib at 1 μM were used for combination studies. DMSO (final concentration 0.5%) was used as control. *p < 0.05, compared to control or single drug alone. (DOC 1147 kb) [file 12871_2017_423_MOESM1_ESM.doc]

**Propofol enhances BCR-ABL TKIs’ inhibitory effects in chronic myeloid leukemia through Akt/mTOR suppression**

Zhimin Tan, Aixia Peng, Jingwen Xu, Mingwen Ouyang

**Supplementary Fig.S1. Expression levels of p-Akt and total Akt in CML cells transfected with p-myr Akt or p-vector in the presence or absence of propofol.** No significant change on Akt phosphorylation level by propofol in K562 cells transfected with constitutively active Akt plasmid. Increased expression level of Akt is shown in p-myr Akt than p-vector cells. Representative western blot photos were shown.

**Supplementary Fig.S2. The combinatory effects of rapamycin and imatinib in CML cells.** Combination of rapamycin and imatinib results in significant more proliferation inhibition and apoptosis induction than rapamycin or imatinib alone in KBM-7, K562 and KU812 cells. Rapamycin at 1 µM and imatinib at 1 µM were used for combination studies. DMSO (final concentration 0.5% ) was used as control. *p<0.05, compared to control or single drug alone.
